# Artificial intelligence for lung ultrasound interpretation: a systematic review

**DOI:** 10.3389/fradi.2026.1899103

**Published:** 2026-07-29

**Authors:** Julia López-Canay, Alberto Fernández-Villar, Cristina Ramos-Hernández, Alberto Fernández-García, Maribel Botana-Rial, Manuel Casal-Guisande

**Affiliations:** 1 NeumoVigo I+i Research Group, Galicia Sur Health Research Institute (IIS Galicia Sur), SERGAS-UVIGO, Vigo, Spain; 2 Pulmonary Department, Hospital Álvaro Cunqueiro, Vigo, Spain; 3 Centro de Investigación Biomédica en Red, CIBERES ISCIII, Madrid, Spain; 4 Department of Functional Biology and Health Sciences, University of Vigo, Vigo, Spain; 5 Diagnostic Imaging Department, Hospital Ribera Povisa, Vigo, Spain; 6 Department of Design in Engineering, University of Vigo, Vigo, Spain

**Keywords:** artificial intelligence, deep learning, lung ultrasound, medical imaging, saliency maps, segmentation, object detection

## Abstract

**Context and objectives:**

Lung ultrasound (LUS) is a safe low-cost tool that enables diagnosis, monitoring and guidance for interventional procedures at the patient's bedside. However, its expansion is hindered by a lack of training programs and the inherent difficulty of interpreting ultrasound images. In this context, Artificial Intelligence (AI) is emerging as a supportive tool for LUS interpretation, ensuring diagnostic efficacy and mitigating the shortage of experts. This systematic review aims to summarize and analyze recent advances in AI-based tools to support LUS interpretation.

**Methods:**

A systematic literature search was conducted across Web of Science, IEEE Xplore, and PubMed databases to identify peer-reviewed original journal articles published between 2015 and November 2025 that employed AI for the identification and localization of lung artifacts, anatomical structures, and pathological findings. Risk of bias was assessed using PROBAST + AI.

**Results:**

Twenty-four studies were included, identifying three main strategies: segmentation (10 studies), object detection (4 studies), and the generation of visual explanations through saliency maps (10 studies). All employed CNN-based architectures. The evaluation metrics used were heterogeneous. The PROBAST + AI assessment showed relevant risk-of-bias concerns, mainly concentrated in the participants and analysis domains.

**Conclusions:**

The development of AI systems to support LUS interpretation shows high potential; however, current studies exhibit significant heterogeneity in their objectives, methodologies, and evaluation metrics. It is necessary to move towards solutions designed for specific clinical environments and to adopt standardized protocols and evaluations that facilitate their implementation in clinical practice.

**Systematic Review Registration:**

https://www.crd.york.ac.uk/PROSPERO/view/CRD420261322517, PROSPERO CRD420261322517.

## 1 Introduction

In recent years, ultrasound has emerged as an essential tool for diagnosing respiratory pathologies, guiding interventional procedures, and monitoring therapeutic response (1). Unlike chest radiography (x-ray) or computed tomography (CT), lung ultrasound (LUS) enables rapid and safe bedside assessment using low-cost portable devices (2, 3). Although its use initially stood out in specific specialized fields such as radiology or emergency medicine, its application in other specialized clinical settings, such as pulmonology, is no longer merely anecdotal (4, 5).

LUS interpretation is based on the identification of anatomical findings and pulmonary artifacts. The latter arise from the difference in acoustic impedance between the pleura (tissue) and the lung (air/fluid) (6). Under normal conditions, an air-filled lung generates reverberation artifacts at the pleural line, resulting in horizontal hyperechoic artifacts known as A-lines (6–9). Likewise, the movement between the visceral and parietal pleura results in lung sliding, producing a “seashore sign” visible on M-mode imaging (8, 9). On the contrary, the absence of lung sliding generates a “barcode sign” (6, 8, 10, 11). Moreover, various pathological conditions lead to reduced lung aeration, which appears on ultrasound images as vertical hyperechoic artifacts known as B-lines. The presence of three or more B-lines is considered clinically relevant and may indicate conditions such as interstitial disease, pulmonary congestion, and heart failure (12–14). Further loss in air content leads to the appearance of pulmonary consolidations (11), characterized by hypoechoic tissue areas and punctate hyperechoic images (10, 15), suggesting the presence of pneumonia and other conditions such as bronchoalveolar carcinoma (16). Finally, the presence of pleural effusions is characterized by the appearance of an anechoic or hyperechoic layer in between pleural layers (17).

Despite its great utility, LUS expansion faces certain challenges in various clinical environments. Ramos-Hernández et al. (18) conclude that, although 91.3% of pulmonologists use LUS for the identification and assessment of pleural effusions, its use in other applications remains limited due to the lack of specific and formal training, with only 5.7% of professionals considered experts (18). In primary care, this adoption is further impeded by strict time limitations and the absence of a standardized pedagogical framework for general practitioners (19). Likewise, the inherent difficulty in interpreting ultrasound images, whose quality is influenced by the operator's experience, patient's anatomy, and equipment settings, results in a labor-intensive procedure with high interobserver variability (20).

In this context, Artificial Intelligence (AI)-based tools can help mitigate the impact of these barriers and ensure the use of LUS in areas like pulmonology, primary care or cardiology. Deep learning (DL) models, specifically convolutional neural networks (CNNs), have demonstrated remarkable success in the analysis of ultrasound images (21). However, while general LUS applications and disease-specific AI models have been reviewed previously (22–26), no systematic review has yet evaluated DL models that visually explain and guide bedside LUS interpretation.

This systematic review aims to summarize and analyze recent advances in AI-based tools for the identification and localization of pulmonary artifacts, anatomical structures and pathological findings to support LUS interpretation, identify key trends and limitations, highlight gaps in the literature and propose a framework for the development of new tools to facilitate their implementation in clinical practice.

## 2 Materials and methods

### 2.1 Study design

A systematic review of the literature was conducted with the aim of providing a summary of the use of AI-based tools for the detection and localization of pulmonary artifacts, anatomical structures and pathological findings in LUS. Additionally, trends and limitations in the literature were identified to provide a framework that encourages future research in this field.

This study was conducted following the Preferred Reporting Items for Systematic Reviews and Meta-Analyses (PRISMA) (27) guidelines. The protocol is available at PROSPERO with Record ID CRD420261322517.

### 2.2 Inclusion and exclusion criteria

Peer-reviewed journal articles published between 2015 and 2025 in English or Spanish, with full text available, were included. Studies were required to describe the development of AI tools for the detection and localization of pulmonary artifacts, anatomical structures, or pathological findings in images or videos of LUS scans performed on humans.

Studies that did not explicitly apply AI techniques or did not detail the development and architecture of the tool were excluded. Additionally, studies limited solely to classification or quantification tasks, those that did not specify the type of artifact identified, or those using data from pediatric patients, animals, or synthetic sources were also excluded.

### 2.3 Search strategy

A comprehensive search was conducted in Web of Science, PubMed, and IEEE Xplore databases to identify articles using AI for the detection and localization of pulmonary artifacts, anatomical structures, and pathological findings to support LUS interpretation. The search strategy employed the following keywords: (“Artificial Intelligence” OR “Machine Learning” OR “Deep Learning” OR “Neural Networks” OR “Automated Analysis” OR “Automated Detection” OR “Intelligent System”) AND (“Lung Ultrasound” OR “Pulmonary Ultrasound” OR “Lung Ultrasonography” OR “Chest Ultrasound” OR “Thoracic Ultrasound”) AND (“B-Lines” OR “A-Lines” OR “Lung Sliding” OR “Pleural Line” OR “Pleural Artifacts” OR “Ultrasound Artifacts” OR “Pleural Effusion”). The search was limited to articles published between January 2015 and November 25, 2025.

After manually removing duplicates, study selection was carried out in two stages: first, by screening titles and abstracts, and subsequently, by reviewing the full text. This process was conducted by two independent reviewers (J.L.-C. and M.C.-G.). In case of disagreement, a third reviewer (A.F.-V.) was consulted.

### 2.4 Synthesis of the results

Data extracted from the studies were organized into four summary tables highlighting key methodological features and main results. The aspects analyzed included study location, data origin and source, sample size, equipment and probes used, and labelling protocol. Additionally, information on the AI model employed, input data, identified elements, validation and split strategy, and the most representative performance evaluation metrics according to the type of study were reported. These metrics included the Dice Similarity Coefficient (DSC), the Jaccard Index or Intersection over Union (IoU), mean Average Precision (mAP), sensitivity (recall), specificity, precision, and the area under the ROC curve (AUC).

Given the obtained results, conducting a meta-analysis was not possible due to the heterogeneity among the included studies in terms of data, methodologies, identified artifacts, and model evaluation metrics.

### 2.5 Risk of bias

Risk of bias and applicability were assessed using the PROBAST + AI tool (28), which extends the Prediction model Risk of Bias Assessment Tool to studies involving AI-based prediction models. Each included study was evaluated across the main methodological domains covered by the tool, including participants, predictors or input data, outcome or reference standard, and analysis.

The assessment considered aspects such as the representativeness of the study population, data source and sample size, adequacy of the labelling or reference standard, patient-level data splitting, validation strategy, mitigation of overfitting, handling of class imbalance, and reporting of appropriate performance metrics. Model development was assessed in terms of methodological quality and applicability, while model evaluation was assessed in terms of risk of bias and applicability.

The risk of bias assessment was performed independently by two reviewers (J.L.-C. and M.C.-G.). Any disagreements were resolved by consensus, and when necessary, a third reviewer (A.F.-V.) was consulted.

## 3 Results

### 3.1 Study selection

A total of 278 articles were identified (112 in Web of Science, 87 in IEEE Xplore, and 78 in PubMed), in addition to one article identified manually. After removing duplicates, 194 articles remained.

**Figure 1:**
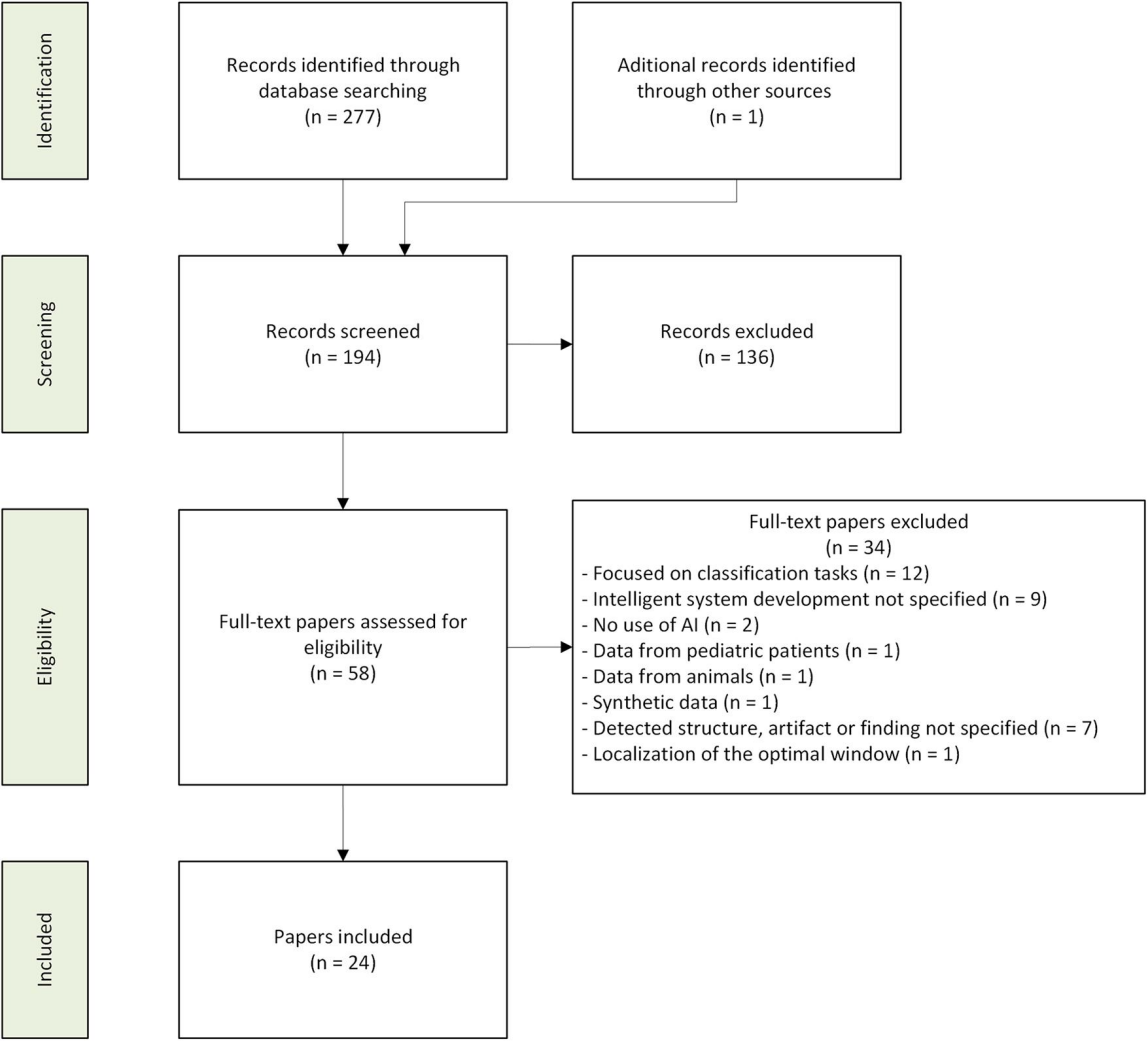
PRISMA flow diagram of the study selection process.

Of these, 58 were selected for full-text review, of which 24 were included in the systematic review. The selection process is detailed in Figure 1, following the PRISMA methodology (27).

### 3.2 Characteristics of the included studies

The included studies, whose main descriptive characteristics are shown in Table 1, were published between 2020 and 2025, covering a wide geographic distribution. The common objective of these studies was the development of intelligent support systems for LUS interpretation, based on the detection and localization of pulmonary artifacts, anatomical structures, and pathological findings.

**Table 1:** Descriptive characteristics of the studies.

Author	Country	Study population	Data source/setting	Sample size	No. of devices	Probe type	Scanning protocol	Labelling protocol
Howell et al. (29)	United Kingdom (UK)	41 patients with COVID-19 pneumonia	Multi-center: 8 hospitals (Leeds Hospitals Trust, UK)	57 images	–	–	–	Manual (2 expert sonographers; cross-verified)
Frank et al. (17)	Israel	33 patients (confirmed/suspected COVID-19; healthy controls)	Multi-center: ICLUS (30) database (5 medical centres, Italy)	2,154 images	3	Lineal and convex	–	Manual pixel-level annotation (expert; verified)
Muñoz et al. (31)	Spain	30 patients	Single-center: ULTRACOV (32) clinical trial	689 videos	1	Convex	12-zone	Hybrid video/frame-level annotation (manual video-level annotation by 1 expert with live marking and offline verification; semi-automated frame-level pixel-level annotation using automated algorithms for pleura/A-lines/B-lines and optical flow tracking for consolidations)
Uchida et al. (33)	Japan	–	Single-center: Jichi Medical University Hospital (Japan)	10,140 images	1	Lineal and convex	–	Manual pixel-level annotation (ultrasound medical specialists + ImageJ software)
Abbasi et al. (34)	Canada	Patients with COVID-19	Single-center: Puerta de Hierro University Hospital (Spain)	4,599 images	1	Phased array and convex	–	–
Lucassen et al. (35)	United States of America (USA)	113 patients with flu-like symptoms	Single-center: ED (Brigham and Women's Hospital, USA)	719 videos; 10,755 images	1	Convex	BLUE (36)	Manual video/pixel-level annotation (manual video-level annotation by 2 LUS experts via consensus decision; manual pixel-level point-of-origin annotation by 1 expert on every fourth frame of positive videos)
Jašcur et al. (37)	Slovakia	48 post-thoracic surgery patients	Single-center: Thoracic Surgery (Jessenius Faculty of Medicine, Slovakia)	545 videos; 6,400 images	1	Lineal	4-zone	Hybrid frame/pixel-level annotation (manual frame-level bounding box annotation by 80 trained volunteers + labelImg software; semi-automated pixel-level semantic segmentation via computer vision algorithms using Otsu's intensity thresholding and morphological dilation)
Vukovic et al. (38)	Australia	24 patients with pleural effusion (39)	Single-center: Internal Medicine (Royal Melbourne Hospital, Australia)	51 videos; 3,041 images	1	Phased array	iLungScan (40)	Manual frame-level polygon annotation (2 independent LUS experts + modified Labelme software)
Xing et al. (41)	China	31 patients with COVID-19 pneumonia	Multi-center: Wuhan Huoshenshan Hospital (China) and Zhejiang University School of Medicine	740 videos; 3,770 images	1	Convex	12-zone	Manual pixel-level annotation (experienced clinicians)
Tan et al. (42)	Singapore	76 dialysis patients (60 hemodialysis; 16 peritoneal dialysis)	Single-center: Nephrology (Khoo Teck Puat Hospital, Singapore)	1,385 images	1	Convex	8-zone	Manual frame-level outline annotation (VGG Image Annotator software)
Xing et al. (43)	China	61 patients (31 COVID-19 pneumonia; 30 pneumothorax)	Multi-center: Wuhan Huoshenshan Hospital (China) and Zhejiang University School of Medicine	4,930 images	2	Convex and lineal	–	Manual (2 expert clinicians)
Bottino et al. (44)	Italy	46 patients	Single-center: Vito Fazzi Hospital; Butterfly and GrepMed platforms	386 images	1	Convex	14-zone	Manual frame-level rectangular ROI annotation (1 expert sonographer)
Tripathi et al. (45)	India	40 patients (hypertension, diabetes, cardiovascular disease, fever, dyspnoea and weakness)	Multi-center: Govern Medical College Kottayam Hospital (India) and Puerta de Hierro University Hospital (Spain)	230 videos	4	Phased array and convex	–	Manual pixel-level annotation (3 clinicians + Label Studio software)
Joseph et al. (46)	India	100 patients with COVID-19	Multi-center: Puerta de Hierro University Hospital (Spain); Sree Chitra Tirunal Institute of Medical Sciences and Technology (India) and Government Medical College (India)	1,200 videos	3	Phased array and convex	14-zone; 12-zone and 16-zone	Manual bounding-box annotation (clinicians + labelImg software)
van Sloun et al. (47)	Netherlands	10 patients	Single-center: CEAVNO study	12 videos; 4,218 images	1	Lineal	–	Manual frame-level binary classification (1 expert)
Chaudhary et al. (48)	Canada	785 patients	Multi-center: ED, ICU and Internal Medicine (2 third level hospitals, Canada)	1,664 videos; 313,109 images	4	Phased array and convex	BLUE (36)	Manual clip-level annotation (1 medical student and 1 family medicine resident + Labelbox; expert verification and discrepancy resolution by 2 expert clinicians)
VanBerlo et al. (49)	Canada	738 patients	Multi-center: ED, ICU and Internal Medicine (2 third level hospitals, Canada)	3,075 videos	3	Phased array, convex and lineal	–	Manual clip-level classification (clinicians and trainees + Labelbox)
Kolarik et al. (50)	Slovakia	48 patients	Single-center: Thoracic Surgery Department (Jessenius Faculty of Medicine, Slovakia)	48 videos	–	Lineal	–	Manual video-level classification (clinicians)
Ni et al. (51)	China	3,966 patients	Single-center: Shanghai Pulmonary Hospital (China)	5,267 images	–	–	6-zone	Manual image-level annotation (2 experienced sonographers)
Chen et al. (52)	China	3,966 patients	Single-center: Ultrasound Department (Shanghai Pulmonary Hospital, China)	5,545 images	1	Convex	6-zone	Manual image-level annotation (2 experienced sonographers, independent blinded review, discordant images discarded)
Arntfield et al. (53)	Canada	–	Multi-center: ED, ICU and Wards (Western University Qpath E database and University of Ottawa Qpath archives)	1,276 videos	3	Phased array, convex and lineal	–	Manual clip-level annotation (Internal data: clinical team members + Labelbox with review by 1 LUS expert; External data: 2 LUS experts + Labelbox via independent blinded review)
Huang et al. (54)	China	31 patients with COVID-19	Single-center: ICU (Wuhan Huoshenshan Hospital, China)	2,062 images	1	Convex	12-zone	Manual image-level annotation (2 experienced clinicians, independent blinded review, discordant images discarded)
Pare et al. (55)	USA	90 patients treated for acute heart failure	Single-center: CRUSH study	716 videos	1	Phased array	8-zone	Manual frame/clip-level annotation (manual frame-level annotation by 1 of 2 ultrasound fellowship-trained physicians; manual clip-level evaluation by 1 expert reviewer blinded to algorithm results; discrepancy resolution via sequential review by up to 3 experts and consensus decision)
Erfanian Ebadi et al. (56)	Canada	300 patients	Multi-center	1,530 videos; 287,549 images	–	–	–	Manual video-level classification (expert radiologists and clinicians)

There is substantial variability in the data used across studies. Among the 21 studies that reported participant numbers, sample sizes ranged from 10 to 3,966 patients. Also, cohort characteristics were heterogenous, with COVID-19 being the most prevalent condition (17, 29–54).

Common clinical settings were the emergency department (ED) [4 studies (35–53)], intensive care unit (ICU) [4 studies (48–54)], and internal medicine [3 studies (38, 48, 49)]. Other settings included thoracic surgery (37, 50), nephrology (42), and general hospital settings.

A marked heterogeneity was observed in sample handling: some studies extracted frames from LUS videos, while others worked directly with LUS images. In terms of origin, 14 studies used single-center data and 10 were multicenter.

The recurrent use of shared databases was identified. Researchers used data from Puerta de Hierro University Hospital (Spain) in 3 studies (34, 45, 46). Similarly, Wuhan Huoshenshan Hospital (China) also provided data for 3 studies (41, 43, 54). Other collaborations included Shanghai Pulmonary Hospital (51, 52), Jessenius Faculty of Medicine in Slovakia (37, 50), and two Canadian tertiary hospitals (48, 49).

Regarding the equipment, most studies used a single ultrasound device; however, some studies utilized data from up to four different devices. The type of probe was specified in 21 studies, ranging from convex, linear, to phased array probes, with 9 studies using multiple probe types.

The labelling protocols varied significantly across the reviewed studies in terms of granularity, quality control, and automation. Annotation scales ranged from highly detailed pixel-level and object-level segmentation to broader frame, video, or clip-level classifications. Quality control measures also diverged, with some studies relying solely on a single expert's interpretation, while others implemented rigorous multi-expert consensus. Furthermore, 2 studies (31, 37) utilized a semi-automated strategy to accelerate the annotation process.

Finally, among the 13 studies reporting a protocol, the 6-zone BLUE protocol (36) was the most common, employed in 2 studies (35, 48), while two others (51, 52) also used a 6-zone scan. Other studies reported scans of 16, 14, 12, 8 and 4 zones.

### 3.3 Main results

Three approaches were identified for AI-assisted identification and localization of pulmonary artifacts, anatomical structures, and pathological findings: image segmentation (17, 29–42), object detection (43–46), and saliency maps (47–55). The results for each category are summarized below.

#### 3.3.1 Image segmentation studies

This category comprises 10 articles (17, 29–42), summarized in Table 2. All studies employed CNNs, with U-Net (59) predominating in 7 studies (29, 31, 33–35, 37, 38). This architecture, originally developed for biomedical applications but now widely adopted across multiple domains, employs a symmetric encoder–decoder structure that enables effective feature extraction and precise localization for semantic segmentation.

**Table 2:** Summary of segmentation studies.

Author	Model	Input	Classes	Validation	Split strategy	DSC	IoU
Howell et al. (29)	Lightweight U-Net	B-mode LUS images 256 × 256 × 1	(1) Background/(2) Ribs/(3) Pleural line/(4) Pulmonary consolidation/(5) Simple pleural effusion/(6) Complex pleural effusion	Internal	90% (51 images) train; 10% (6 images) test	0.20	–
Frank et al. (17)	DeepLabV3++ (57) (ResNet-50)	3 channel images: (1) B-mode LUS image (2) Vertical artifacts mask (3) Signed distance to pleural line mask	(0) Continuous pleural line and A-lines/(1) Pleural line alterations and few B-lines/(2) B-lines and small consolidations/(3) B-lines and big consolidations/(4) Background	Internal	Patient level: ∼74% (1,601 images) train; ∼26% (553 images) test	0.70	–
Muñoz et al. (31)	Attention U-Net (58)	B-mode LUS images 256 × 128	(1) Pleural line/(2) Background	Internal	Patient level: 90% (27 patients, 9,159 images) train (70% images train/30% images test); 10% (3 patients) second validation	0.83	0.72
			(1) A-lines/(2) Background			0.40	0.56
			(1) B-lines/(2) Background			0.87	0.84
			(1) Consolidations/(2) Background			0.96	0.95
Uchida et al. (33)	U-Net	B-mode LUS images 240 × 240 × 1	(1) Pleural/(2) Background	Internal	∼98.6% (10,000 images) train; ∼1.4% (140 images) test	0.99	0.98
Abbasi et al. (34)	TransBound-UNet	B-mode LUS images	(1) A-lines/(2) B-lines/(3) Background	Internal	5-fold cross-validation	0.8	0.73
Lucassen et al. (35)	EfficientNet-18 + U-Net	B-mode LUS images 384 × 256	(1) B-lines origin/(2) Background	Internal	Patient level: 80% (1,112 videos) train with 5-fold cross-validation; 20% (307 videos) test	–	–
Jašcur et al. (37)	U-Net	B-mode LUS images 480 × 480	(1) Lung/(2) Background	Internal	50% (3,200 images) train; 25% (1,600 images) validation; 25% (1,600 images) test	–	0.75
			(1) Pleural line/(2) Background			–	0.61
			(1) Rib shadow/(2) Background			–	0.81
Vukovic et al. (38)	STN + U-net	B-mode LUS images 806 × 550	(1) Background/(2) Pleural effusion	Internal	Patient level: ∼67% (16 patients, 1,831 images) train; ∼12% (3 patients, 610 images) validation; ∼21% (5 patients, 600 images) test	0.7	–
Xing et al. (41)	Three-level cascaded encoder–decoder model based on convolution and multilayer perceptron (MLP)	ROI pleural line images	(1) Pleural line/(2) Background	External	Patient level: ∼87.7% (31 patients, 1,420 images) train with 5-fold cross-validation; ∼12.3% (25 patients, 200 images) test	0.87	–
Tan et al. (42)	Mask-RCNN and YOLACT	B-mode LUS images	(1) B-lines/(2) Background	Internal	Patient level: ∼80.2% (61 patients, 1,003 images) train; ∼19.8% (15 patients, 382 images) test	–	–

Two studies (33, 37) opted for the conventional architecture, while others employed optimized variants. Howell et al. (29) implemented a lightweight U-Net with fewer filters, LeakyReLU activations, and bilinear upsampling for faster processing. Meanwhile, Muñoz et al. (31) employed Attention U-Net (58), integrating attention gates to focus on target structures, thereby optimizing sensitivity and precision.

Other innovations in this field include the use of Vision Transformers (ViT). Abbasi et al. (34) designed TransBound-UNet, a lightweight version of the TransUNet (60) architecture. Similarly, Lucassen et al. (35) employed EfficientNet-18 in the encoder, while Vukovic et al. (38) combined U-Net with a Spatial Transformer Network (STN) to optimize segmentation by mapping regions of interest (ROIs).

Additional innovations include the use of the DeepLabV3++ architecture (57) by Frank et al. (17), aimed at addressing the loss of spatial resolution through *atrous* convolutions, and the implementation of hybrid models such as Mask R-CNN and YOLACT by Tan et al. (42) to achieve real time instance segmentation. Finally, Xing et al. (41) developed a three-level cascaded encoder–decoder architecture.

Eight studies employed grayscale B-mode images as input (29, 31, 33–35, 37, 38, 42). A notable exception is the work by Frank et al. (17), who used a three-channel input enriched with vertical artifact masks and pleural distance maps. Additionally, Xing et al. (41) preprocessed the images by extracting a ROI using a Faster R-CNN (61) prior to segmentation.

Studies applied both binary and multiclass approaches. Among the latter, highly complex models are presented, such as that of Howell et al. (29), where up to six different classes were identified, distinguishing background, ribs, pleural line, pulmonary consolidation, and simple and complex pleural effusion. Meanwhile, Frank et al. (17) proposed a four-level categorization differentiating background, continuous pleural line with A-lines, pleural alterations with few vertical artifacts, and two severity levels for the presence of B-lines and consolidations (minor and major). Finally, Abbasi et al. (34) focused on multiclass segmentation of pulmonary artifacts, distinguishing A-lines, B-lines, and background.

On the other hand, binary approaches either used multiple independent segmentation models or targeted a single landmark or artifact. In the latter, pleural line segmentation stands out, addressed in two studies (33, 41).

Finally, the validation strategies exhibited considerable heterogeneity. Six studies (17, 31, 35, 38, 41, 42) ensured patient-level splits to avoid bias while one study (41) conducted an external validation process. Also, 5-fold cross-validation was the most used split strategy (34, 35, 38, 41). Performance was mainly assessed using the DSC and the IoU index. The DSC was reported in seven studies (17, 29–41) with values ranging from 0.2 to 0.99, while IoU ranged from 0.61 to 0.98 in four studies (31, 33, 34, 37).

#### 3.3.2 Object detection studies

This group includes 4 articles (43–46), summarized in Table 3, that implement object detection strategies to support LUS interpretation. All approaches are based on CNNs, with two dominant architectures: single-stage networks and region-based networks (R-CNNs).

**Table 3:** Summary of object detection studies.

Author	Model	Input	Classes	Validation	Split strategy	mAP (IoU 0.5)	Sensitivity	Precision
Xing et al. (43)	Faster R-CNN (ResNet-50)	B-mode LUS images	Pleural line bounding box	External	Patient level: ∼64% (39 patients, 3,000 images) train with 5-fold cross-validation; ∼36% (22 patients, 1,930 images) test	–	–	–
Bottino et al. (44)	YOLO (CSPDarkNet53)	B-mode LUS images 224 × 224	B-lines bounding box	Internal	80% (309 images) train; 20% (77 images) 5-fold cross-validation	–	81.0%	92.0%
Joseph et al. (46)	YOLOv5s (62)	B-mode LUS images 416 × 416	Bounding box: (1) pleura/(2) ribs/(3) shadow/(4) A-lines/(5) B-lines/(6) B-patches/(7) Pulmonary consolidations/(8) Air bronchograms	Internal	∼70% (570 images) train; ∼20% (163 images) validation; ∼10% (83 images) test	66.0%	68.5%	67.0%
Tripathi et al. (45)	KeyNet	LUS image pairs 256 × 256 × 10: 10 channels generated by the RT-FPM acoustic feature map (5 for horizontal features + 5 for vertical features)	10 keypoints: (i) Pleura (ii) A-lines (iii) B-lines	Internal	1,024 image pairs train; 512 image pairs validation; 1,000 images test	–	99.0%	83.0%

Within single-stage architectures, YOLO (You Only Look Once) (63) networks were used in 2 studies (45, 46). These networks treat object detection as a single regression problem, enabling the simultaneous prediction of bounding boxes and class probabilities through global image processing. This makes them highly efficient, facilitating their integration into applications requiring real-time processing. Regarding the variants used, Bottino et al. (44) employed a pretrained CSPDarkNet53 as the backbone, while Joseph et al. (46) used YOLOv5s (62), an optimized version to improve speed and accuracy.

As an alternative, the use of R-CNNs (64) was identified. This approach operates in two stages: the identification of ROIs and their subsequent classification using CNNs. Specifically, Xing et al. (43) implemented a Faster R-CNN (61), which incorporates a Region Proposal Network (RPN) to speed up processing and ensure efficient execution in clinical settings. Additionally, the approach by Tripathi et al. (45) stands out, as they employ an architecture for keypoint detection.

Regarding the scope of detection, proposals with different levels of complexity were identified. On one hand, 2 studies (43, 44) developed models aimed at detecting a single element. In contrast, Joseph et al. (46) and Tripathi et al. (45) implemented multiclass approaches.

Finally, model evaluation showed heterogeneity in validation strategies and reported metrics. Only one study (43) employed an external validation process. Two studies (43, 44) used 5-fold cross-validation, with Xing et al. (43) implementing a patient-level data split. In terms of metrics, precision and sensitivity emerged as the primary indicators in 3 studies (44–46), with precision ranging from 68.5% to 99.0% and sensitivity from 67.0% to 92.0%. It is worth noting that the mAP, a standard metric in object detection, was reported only by Joseph et al. (46), who achieved a value of 66.0% using an IoU threshold of 0.5.

#### 3.3.3 Studies that employed saliency maps

This group includes 10 studies (47–55), summarized in Table 4. The primary focus of these studies lies in building classification models for detecting artifacts, anatomical structures, and pathological findings using CNNs to support LUS interpretation. The main mechanism, the CNN, does not directly detect the location of a finding in a specific area of the image; rather, it indicates whether the feature is present or not, performing a classification. The significance of these approaches lies in the integration of visual explainability mechanisms based on saliency maps, which allow the identification of specific regions of the image that underlie the model's prediction.

**Table 4:** Summary of studies that employed saliency maps.

Author	Model	Input	Classes	Validation	Split strategy	AUC	Sensitivity	Specificity
van Sloun et al. (47)	CNN + Grad-CAM	B-mode LUS images 256 × 352 × 1	(1) B-lines presence/(2) B-lines absence	Internal	67% (8 videos) train; 33% (4 videos) 3-fold cross-validation	0.874	85.6%	69.7%
Chaudhary et al. (48)	CNN (EfficientNetB0) + Grad-CAM	B-mode LUS images	(1) Pleural effusion presence/(2) Pleural effusion absence	Internal	Patient level: 85% (668 patients, 266,670 images) train with 10-fold cross-validation; 15% (117 patients, 46,439 images) test	0.939	85.9%	89.3%
VanBerlo et al. (49)	CNN (EfficientNetB0) + Grad-CAM	M-mode LUS images 180 × 224	(1) Lung sliding presence/(2) Lung sliding absence	Internal	Patient level: 85% (614 patients, 2,535 videos) train with 10-fold cross-validation; 15% (124 patients, 540 videos) test	0.973	93.5%	87.3%
Kolarik et al. (50)	3D CNN (Resnet3D-18) + Vanilla Saliency Map (65) and SmoothGrad (66)	B-mode LUS videos 30 × 30 × 3	(1) Lung sliding presence/(2) Lung sliding absence	Internal	Patient level: 70% train; 30% test	–	93.60%	78.53%
Ni et al. (51)	MEVAL CNN (ResNet50) + Grad-CAM	3 channel images 641 × 395 × 3: (1) LUS images (2) Enhanced view via K-means clustering (3) Enhanced view via vertical linear adjustment	(1) A-lines/(2) B-lines/(3) Pulmonary consolidations/(4) Pleural effusion	Internal	Patient level: ∼69% (2,751 patients) train with 5-fold cross-validation; ∼17% (688 patients) active learning test; ∼13% (527 patients) test	0.9989	98.76%	98.6%
Chen et al. (52)	CNN (ResNet) + Grad-CAM	B-mode LUS images	(1) A-lines/(2) B-lines/(3) Pulmonary consolidations/(4) Pleural effusion	Internal	80% (4,436 images) train; 20% (1,109 images) test	0.9976	98.27%	99.41%
Arntfield et al. (53)	CNN (VGG16) + Grad-CAM	B-mode LUS images	(1) A-lines/(2) B-lines	External	Patient level: (408 patients) train with10-fold cross-validation; (276 patients) internal test; (72 patients) external test	0.93	83.0%	82.0%
Huang et al. (54)	CNN (NCA-ResNet) + Grad-CAM	B-mode LUS images 300 × 300	(0) A-lines presence and B-lines absence/(1) B-lines presence (separated 7 mm)/(2) Confluent B-lines presence/(3) Coalescent B-lines presence	Internal	Patient level: 80% (25 patients, 1,735 images) train; 20% (6 patients, 327 images) test	–	90.43%	–
Pare et al. (55)	CNN (ResNet18) + Grad CAM	B-mode LUS images 224 × 224	(0) B-lines absence/(1) 1 or 2 B-lines (each occupies <10% of the field)/(2) > 2 B-lines (each occupies <10% of the field)/(3) B-lines occupy 10–49% of the field/(4) B-lines occupy >50% of the field	Internal	Patient level: - Development (30 patients, 49,952 images) 10-fold cross-validation: 80% train; 10% validation; 10% test - Evaluation (60 patients, 476 videos)	0.967	96.3%	92.4%
Erfanian Ebadi et al. (56)	3DCNN + saliency maps	LUS videos 224 × 224 × 3	(1) A-lines	Internal	80% (1,225 videos) train; 20% (306 videos) 5-fold cross-validation	0.94	91.0%	–
			(2) B-lines			0.91	86.0%	–
			(3) Consolidations and/or pleural effusion			0.96	92.0%	–

Eight studies (47–55) used 2D CNNs combined with gradient-based activation maps (Grad-CAM) (67). On the other hand, 2 studies (50, 56) opted for spatiotemporal approaches using 3D CNNs. Among these, Kolarik et al. (50) specified the construction of activation maps using Vanilla Saliency Maps (65) and SmoothGrad (66).

Most authors used grayscale B-mode LUS images (47, 48, 52–55) as input. However, notable variations were identified: VanBerlo et al. (49) processed M-mode images to assess pleural motion, while Ni et al. (51) proposed a three-channel input combining the original image with two views optimized through K-means clustering and vertical linear adjustment. Finally, studies based on 3D architectures (50, 56) directly used LUS video sequences.

The sample of studies is evenly divided between those that performed binary classification (47–53) and those that opted for multiclass classification (51, 52, 54–56). Within the first group, two studies focused on detecting the absence of lung sliding (49, 50). Two others addressed the presence of B-lines (50, 53). Finally, Chaudhary et al. (48) focused their approach on the detection of pleural effusions.

Among the multiclass models, 2 studies (51, 52) proposed a four-category classification: A-lines, B-lines, pulmonary consolidations, and pleural effusion. Erfanian Ebadi et al. (56) simplified this division with a three-class model that groups consolidations and pleural effusion into a single category. The remaining studies (54, 55) focused on artifact identification.

In terms of model validation, 7 studies (48–55) employed patient-level data splits. Only one study (53) conducted an external validation process. AUC values were reported in 8 studies, ranging from 0.874 to 0.9989. Similarly, sensitivity was reported in all studies, with values between 83.0% and 98.76%, while specificity was reported in eight studies, ranging from 69.7% to 99.41%.

### 3.4 Risk of bias

Table 5 summarizes the quality and applicability of model development, alongside the risk of bias and applicability of model evaluation, assessed using the PROBAST + AI (28) tool. Overall, the included studies exhibited low quality and a high risk of bias. While the predictors and outcome domains generally showed high quality and low risk of bias, the primary deficiencies were concentrated within the participants and analysis domains.

**Table 5:** Model development quality and applicability and model evaluation risk of bias and applicability of the included studies according to PROBAST + AI (28) (1. participants, 2. predictors, 3. outcome, 4. analysis).

Study	Model development							Model evaluation							Overall			
	Quality				Applicability			Risk of bias				Applicability			Model development		Model evaluation	
	1	2	3	4	1	2	3	1	2	3	4	1	2	3	Quality	Applicability	Risk of bias	Applicability
Howell et al. (29)	−	+	+	−	+	+	+	−	+	+	−	+	+	+	−	+	−	+
Frank et al. (17)	+	+	+	+	+	+	+	+	+	+	+	+	+	+	+	+	+	+
Muñoz et al. (31)	−	+	−	−	−	+	+	−	+	−	−	−	+	+	−	−	−	−
Uchida et al. (33)	−	+	+	−	?	+	+	−	+	+	−	?	+	+	−	?	−	?
Abbasi et al. (34)	−	+	+	−	?	+	+	−	+	+	−	?	+	+	−	?	−	?
Lucassen et al. (35)	+	+	+	+	+	+	+	+	+	+	+	+	+	+	+	+	+	+
Jašcur et al. (37)	+	+	−	+	+	+	+	+	+	−	−	+	+	+	−	+	−	+
Vukovic et al. (38)	−	+	+	−	+	+	+	−	+	+	−	+	+	+	−	+	−	+
Xing et al. (41)	−	+	+	−	+	+	+	−	+	+	−	+	+	+	−	+	−	+
Tan et al. (42)	−	+	+	−	+	+	+	−	+	+	−	+	+	+	−	+	−	+
Xing et al. (43)	−	+	+	−	+	+	+	−	+	+	−	+	+	+	−	+	−	+
Bottino et al. (44)	−	+	+	−	+	+	+	−	+	+	−	+	+	+	−	+	−	+
Tripathi et al. (45)	−	+	+	−	+	+	+	−	+	+	−	+	+	+	−	+	−	+
Joseph et al. (46)	−	+	+	−	+	+	+	−	+	+	−	+	+	+	−	+	−	+
van Sloun et al. (47)	−	+	+	−	+	+	+	−	+	+	−	+	+	+	−	+	−	+
Chaudhary et al. (48)	+	+	+	+	+	+	+	+	+	+	+	+	+	+	+	+	+	+
VanBerlo et al. (49)	+	+	+	+	+	+	+	+	+	+	+	+	+	+	+	+	+	+
Kolarik et al. (50)	−	+	+	−	+	+	+	−	+	+	−	+	+	+	−	+	−	+
Ni et al. (51)	+	+	+	+	+	+	+	+	+	+	+	+	+	+	+	+	+	+
Chen et al. (52)	+	+	+	+	+	+	+	+	+	+	−	+	+	+	+	+	−	+
Arntfield et al. (53)	+	+	+	+	+	+	+	+	+	+	+	+	+	+	+	+	+	+
Huang et al. (54)	−	+	+	−	+	+	+	−	+	+	−	+	+	+	−	+	−	+
Pare et al. (55)	−	+	+	−	+	+	+	−	+	+	+	+	+	+	−	+	+	+
Erfanian Ebadi et al. (56)	+	+	+	+	+	+	+	+	+	+	−	+	+	+	+	+	−	+

+, Favorable judgment (high quality or applicability, or low risk of bias); ?, unclear judgement; −, unfavorable judgment (low quality or applicability, or high risk of bias).

Key deficiencies in the participants domain included critically small sample sizes, the inclusion of patients with only a specific disease, and an over-reliance on shared and hand-selected databases. Regarding the analysis domain, key deficiencies included failing to perform patient-level data splits, missing standard computer vision metrics, ineffective mitigation of overfitting, and improper handling of class imbalances.

Conversely, studies generally demonstrated high applicability for both model development and evaluation.

## 4 Discussion

### 4.1 Main findings

To our knowledge, this is the first systematic review specifically focused on AI methods for the detection, localization, and visual interpretation support of LUS findings. This study identified 24 research articles applying AI methods for the detection and localization of pulmonary artifacts, anatomical structures, and pathological findings to aid clinical decision-making in LUS. The identified studies were categorized into three different methodological approaches: semantic segmentation (17, 29–42), object detection (43–46), and classification incorporating visual explainability mechanisms through saliency maps (47–55).

All approaches are based on DL strategies using CNN implementations. In the field of segmentation, the U-Net architecture has become the reference standard, while YOLO-based architectures dominate in object detection. Meanwhile, most models focused on visual explainability employ 2D CNNs coupled with Grad-CAM activation maps.

The studies focused primarily on the localization and detection of B-lines (17, 31–55), A-lines (17, 31–46, 53, 54, 56), and the pleural line (17, 29, 31, 33, 37, 41–46), which are key elements for the diagnosis of various pulmonary conditions.

The performance of segmentation models was evaluated using metrics such as the DSC, reported in seven studies, and the IoU, used in four studies. DSC values ranged from 0.2 to 0.99, while IoU values ranged from 0.61 to 0.98. In object detection studies, precision was reported in three studies, ranging from 67.0% to 92.0%; sensitivity in two studies, ranging from 68.5% to 99.0%, and mAP (with an IoU threshold of 0.5) in one study, with a value of 66.0%. Finally, classification studies reported AUC in eight studies, with values ranging from 0.874 to 0.9989; sensitivity was reported in all studies, ranging from 83.0% to 98.76%; while specificity was reported in eight studies, ranging from 69.7% to 99.41%.

### 4.2 Limitations of the included studies

The studies included in this review present several methodological and design limitations that shape the current landscape of AI-driven LUS interpretation. Primarily, there is notable heterogeneity in the computer vision strategies used to detect and localize features of interest, with three distinct technical approaches currently coexisting. This algorithmic variance is further compounded by variability in the underlying data volume and formats. While some systems process static images or isolated frames extracted from videos, others process full video sequences directly. These discrepancies create a fragmented technical landscape that hinders objective comparisons.

One of the technical approaches relies on the use of saliency maps. While these techniques enhance the visual explainability of DL architectures, it is prone to provide misleading explanations. Furthermore, because models adopting this approach are often evaluated solely on classification performance, the literature frequently lacks a precise, quantitative assessment of their actual localization quality.

Data constraints further undermine the statistical robustness of the reported findings. Sample sizes vary wildly from 10 to 3,966 patients. Although isolated large-scale datasets contain up to 313,109 images, the literature is heavily dominated by small-to-medium cohorts. This reliance on limited sample sizes restricts the ability of these systems to capture the true variance of diverse clinical populations. Additionally, only 3 studies conducted an external validation process, demonstrating that results evaluated solely on internal datasets can mask the true generalizability of the developed models.

Furthermore, data scarcity has led to hidden redundancies across the literature. A rigorous analysis of institutional origins reveals significant dataset overlap, which can be synthesized into five distinct clusters. First, a Canadian cohort (48, 49) shares an overlapping patient registry derived from EDs and ICUs across two tertiary hospitals. Second, a prominent Wuhan cohort (41, 43, 54) relies on the exact same pool of COVID-19 patients from Huoshenshan Hospital. Third, a Chinese institutional database at the Shanghai Pulmonary Hospital is duplicated across two studies (51, 52). Fourth, a Slovakian thoracic surgery cohort utilizes the same 48-patient population from the Jessenius Faculty of Medicine across two papers (37, 50). Finally, a Spanish multi-center node features overlapping patient data sourced from Puerta de Hierro University Hospital across three studies (34, 45, 46). This high degree of data recycling indicates that model development is heavily restricted to specific, non-independent geographic and institutional cohorts.

A large portion of the literature focuses on COVID-19 cohorts, introducing a selection bias that restricts the generalizability of these DL models to other pathologies. This imbalance highlights a gap in the literature: a lack of AI systems designed for heterogeneous, real-world clinical scenarios where patients are affected by different conditions. Furthermore, while the reviewed research spans diverse high-acuity environments, such as EDs, ICUs, internal medicine, and thoracic surgery, there is a lack of data from primary care or outpatient settings, where AI tools could otherwise support routine LUS adoption in daily practice.

Also, there is a lack of standardization in image acquisition protocols and hardware. Previous evidence establishes that acquisition protocols, equipment settings, and transducer types influence clinical outcomes (68, 69). However, the lack of international consensus on LUS performance (1) is reflected in the analyzed literature, which features variable equipment, probes, and scanning protocols. While some investigations rely on data from a single device and a specific probe, others utilize heterogeneous datasets pooled across multiple manufacturers, hindering direct comparisons across models.

Regarding performance evaluation, it is important to highlight the absence of standardized metrics in several studies. This is particularly critical in object detection models, where mAP, a standard metric for such architectures, is frequently omitted. Furthermore, the reported results across validation and test sets are highly heterogeneous, with several algorithms exhibiting suboptimal performance.

Finally, these studies serve primarily as proof-of-concept research. The lack of prospective clinical implementation means that the performance of these tools remains unverified in real-world environments. Consequently, there is currently no prospective evidence comparing AI-assisted clinical performance against standard practice without AI support, which remains a fundamental gap that must be addressed to justify their integration into routine care.

### 4.3 Clinical implications

LUS is a cost-effective and safe tool for real-time assessment of pulmonary conditions. Although its use has increased notably in critical settings such as EDs and ICUs, its integration into other areas, such as pulmonology, primary care or cardiology, remains limited due to a scarcity of specialized training programs. This technological gap has hindered the adoption of LUS in environments where it could be decisive for managing various pathologies, guiding interventional procedures, and monitoring treatment response.

In resource-constrained environments, such as rural communities and low-to-middle-income countries, access to advanced imaging like CT, magnetic resonance imaging (MRI), or x-ray is often limited. While LUS is a highly valuable alternative in these settings, its implementation is frequently prevented by a shortage of experts, who are typically concentrated in tertiary hospitals, and a lack of standardized training. Similarly, while LUS has significant potential for use by emergency medical technicians, nurses, and paramedics to expand diagnostic capabilities in community and peripheral centers, the current lack of widespread training remains a critical barrier.

Moreover, LUS empowers clinicians in fields like pulmonology, primary care, and cardiology to perform bedside imaging, potentially reducing dependence on overloaded radiology departments. This shift toward point-of-care assessment significantly reduces the need for external referrals and improves diagnostic efficiency (70).

In this context, the development of AI tools to support the interpretation of LUS through the detection and localization of artifacts, anatomical structures, and pathological findings represents a crucial step toward facilitating its widespread use. For instance, AI-driven B-line identification offers a more reliable and objective assessment of the lung parenchyma. By generating automated segmentation masks overlaid directly onto the LUS image, segmentation models enable the calculation of complex metrics such as the B-line Artifact Score, which quantifies the exact percentage of each intercostal space occupied by B-lines (29). This metric improves the tracking of respiratory diseases like COVID-19. On a workflow level, the automatic detection and counting of B-lines can accelerate the computation of these standardized LUS scores, potentially reducing both inter-operator variability and reporting time at the bedside. Furthermore, this automated quantification facilitates non-invasive fluid status assessment for hemodialysis and peritoneal dialysis patients (42).

Complex models that allow for the identification and localization of various anatomical structures and artifacts serve as a transverse tool to assess disease severity. By identifying the pleural line, A-lines, and B-lines simultaneously, these systems optimize LUS scanning workflows and automatically calculate diagnostic scores. For instance, automated pleural line localization guides clinicians in correct probe positioning. Furthermore, because underlying respiratory conditions cause distinct structural alterations to the pleura, such as thickening, roughness, or fragmentation, automating its extraction allows for a highly objective grading of lung involvement. When this structural analysis is integrated with the concurrent detection of A-lines and B-lines, it enables the automated computation of standardized COVID-19 lung involvement scores (71).

Finally, pleural effusion segmentation is another highly relevant application. AI-assisted segmentation can aid clinicians during volume estimation, which helps establish a diagnosis and is valuable for the follow-up and monitoring of patients, especially when drainage is not required (72).

#### 4.3.1 Practical implementation and workflow integration

The tools identified in this review will facilitate the expansion of LUS use and ensure its diagnostic efficiency through effective integration into clinical practice. Even though none of the developed algorithms are currently in use or were implemented into a real clinical setting, their future implementation has been discussed across studies.

On one hand, these systems can be implemented directly into ultrasound equipment, providing real-time support and reducing examination time (32). This assistance enables precise localization of pulmonary artifacts, anatomical structures, and pathological findings simultaneously with image acquisition, making the technique more accessible for less experienced clinicians (73).

Likewise, AI-assisted interpretation of the examination can improve diagnostic accuracy and help reduce interpretation errors during LUS examination. In academic and professional settings, these tools can be integrated into specialized training programs, which may enable continuous learner support and offer a potential reduction in the LUS learning curve.

The consolidation of these systems in clinical settings such as pulmonology, primary care or cardiology would help optimize healthcare resource management. By strengthening the diagnostic capability of LUS, it is possible to reduce reliance on other imaging tests that are more costly or involve radiation, such as CT or chest x-ray. Consequently, this could minimize unnecessary escalations to secondary care.

#### 4.3.2 Adoption barriers and enablers

The primary barriers to the adoption of these systems include the difficulty of acquiring high-quality training data. The construction of the datasets requires time and experts to annotate LUS videos and images. This effort is multiplied for the development of segmentation and object detection models, which require high labelling efforts.

Furthermore, the lack of hardware standardization, compounded by the use of diverse commercial devices, probes and configurations, hinders the seamless implementation of AI solutions across different clinical departments. This challenge is exacerbated by the heterogeneity in the literature, as there is no consensus on which elements should be identified to ensure correct LUS interpretation across a wide range of pathologies. Additionally, the reliance on computationally intensive DL models complicates real-time implementation.

Finally, while LUS is inherently a cost-effective tool, the cost-efficiency of AI-augmented workflows remains largely unexplored. Although these tools show potential for clinical impact in resource-constrained environments, objective prospective studies are necessary to quantify their economic value and ensure successful clinical integration.

Facilitating factors for their adoption include access to open databases, as well as the wide variety of available architectures to address this task, enabling selection of the most appropriate ones. Additionally, the ability to achieve precise localization of structures stands out, as it not only facilitates ultrasound interpretation but also enables the development of specialized training programs. This would promote the expansion of LUS use in clinical settings where its application remains limited, reducing the current reliance on more complex diagnostic tests.

### 4.4 Future opportunities

Currently, the clinical application of AI tools for LUS interpretation remains in its early stages. The studies analyzed in this review are primarily in the research and validation phase; while some have undergone external validation, none of the developed algorithms have been implemented in a clinical environment. Consequently, data regarding their performance in real-world clinical settings is currently unavailable. Future research must prioritize the transition from proof-of-concept models to integrated clinical tools.

To successfully scale these algorithms, they require continuous, prospective training. This approach, supported by ongoing human expert supervision, will allow models to adapt to diverse clinical environments, minimize performance drift, and ensure long-term diagnostic reliability in real-world practice.

For successful clinical integration, it is essential to develop lightweight DL models capable of real-time operation to assist clinicians directly during patient examinations and provide instantaneous decision support. Because the main barrier to LUS expansion continues to be its operator dependence, future systems should not be limited to identifying and localizing ultrasound findings but should also actively guide image acquisition. This clinical translation requires a dual framework of automated probe guidance and real-time image quality assessment, which concurrently verify correct probe positioning and evaluate the diagnostic viability of the captured frames. Specifically, the automated localization of foundational anatomical landmarks, such as the pleural line and the rib shadows, can serve as an objective structural reference to confirm appropriate acoustic windowing. Furthermore, these intelligent systems can address common technical factors that degrade image quality, such as motion artifacts caused by unintended probe movement or rapid patient respiration. Seamlessly integrating real-time acquisition feedback into frontline workflows will unlock true bedside utility, facilitating rapid patient assessment, immediate triaging, and efficient disease monitoring.

Likewise, the growing interest in utilizing LUS across specific clinical settings, such as pulmonology, primary care, or cardiology, must be accompanied by studies focused directly on these contexts to ensure that the models remain applicable and effective in diverse clinical routines.

Another key aspect is moving toward a multimodal approach that integrates ultrasound images with relevant clinical information. Combining imaging features with patient data will enable more precise analysis, advance personalized medicine, and improve overall model performance.

Beyond direct clinical care, these tools can be leveraged to establish structured training and learning programs to expand the available pool of LUS experts. By providing interactive, real-time feedback to learners, AI assistants have the potential to shorten training time required for image acquisition (7, 74, 75).

Finally, while current studies are based on single-point diagnoses, there is a significant opportunity to develop algorithms capable of comparing examinations over time. This would allow for automatic and objective quantification of therapeutic response and support the long-term monitoring of multiple respiratory pathologies.

### 4.5 Limitations of this review

This systematic review has several limitations. First, the methodological heterogeneity of the identified studies, together with the small number of investigations within each group and the diversity of reported metrics, hindered the conduct of a meta-analysis. Although the inclusion and exclusion criteria were rigorously defined, it is possible that a relevant article was omitted if its methodology was not described in sufficient detail.

Second, the literature search was limited to publications in Spanish and English, introducing potential language bias. Furthermore, the scope was limited to journal articles, excluding proceedings and conference papers, which may have introduced selection bias. In addition, although many well-recognized databases reflecting the interdisciplinary nature of the topic were used (Web of Science, IEEE Xplore, and PubMed), other sources could have been explored.

Third, the AI models presented in the reviewed studies primarily serve as proofs-of-concept. Although a subset of these studies performed external validation, none of the developed models have been implemented in real-world clinical workflows. This lack of deployment prevented an assessment of the clinical utility, effectiveness, and operational impact of these tools. Consequently, these findings highlight a critical gap in the literature. Future research should transition from technical validation to clinical implementation to demonstrate tangible benefits to patient care.

Finally, although we utilized the PROBAST + AI tool to assess the quality, risk of bias, and applicability of the included studies, a degree of subjectivity is inherent to such evaluations. Integrating complementary assessment tools in future reviews could provide a more robust evaluation and strengthen overall findings.

## 5 Conclusions

This systematic review demonstrates the effectiveness of applying AI techniques to support LUS interpretation. The identified studies are based on DL strategies, revealing three distinct approaches: segmentation, object detection, and activation map generation.

Despite the growing interest in this field, several limitations remain. First, the studies are highly heterogeneous in both their objectives and the methodologies employed. Additionally, there is variability in the reported metrics, and many studies do not perform an appropriate evaluation of model performance using standard metrics.

Future research should focus on developing systems specifically designed for its use in various clinical settings such as pulmonology, cardiology or primary care, where factors such as time constraints and the lack of specialized training are hindering the adoption of LUS. Additionally, it will be necessary to advance towards systems that not only assist in interpretation but also guide the operator, integrate relevant clinical information, and allow for the comparison of studies over time, with the aim of improving diagnostic accuracy and the clinical utility of these systems.

## Data Availability

The original contributions presented in the study are included in the article, further inquiries can be directed to the corresponding author.
